# Co-occurrence of relapsing polychondritis and autoimmune thyroid diseases

**DOI:** 10.1186/s13023-022-02261-5

**Published:** 2022-05-10

**Authors:** Toshiki Nakajima, Hajime Yoshifuji, Yoshihisa Yamano, Kimiko Yurugi, Yasuo Miura, Taira Maekawa, Tsuneyasu Yoshida, Hiroshi Handa, Koichiro Ohmura, Tsuneyo Mimori, Chikashi Terao

**Affiliations:** 1 Department of Clinical Immunology and Rheumatology, Tazuke Kofukai Medical Research Institute, Kitano Hospital, Osaka, Japan; 2 Department of Rheumatology and Clinical Immunology, Kyoto University Graduate School of Medicine, Kyoto, Japan; 3 Department of Rare Diseases Research, Institute of Medical Science, St. Marianna University School of Medicine, Kanagawa, Japan; 4 Division of Neurology, Department of Internal Medicine, St. Marianna University School of Medicine, Kanagawa, Japan; 5 Department of Transfusion Medicine and Cell Therapy, Kyoto University Hospital, Kyoto, Japan; 6 Division of Respiratory and Infectious Diseases, Department of Internal Medicine, St. Marianna University School of Medicine, Kanagawa, Japan; 7 Ijinkai Takeda General Hospital, Kyoto, Japan; 8 Laboratory for Statistical and Translational Genetics, RIKEN Center for Integrative Medical Sciences, Kanagawa, 230-0045 Japan; 9 Clinical Research Center, Shizuoka General Hospital, Shizuoka, Japan; 10 The Department of Applied Genetics, University of Shizuoka School of Pharmaceutical Sciences Graduate School of Pharmaceutical Sciences, Shizuoka, Japan

**Keywords:** Relapsing polychondritis, Autoimmune thyroid disease, Human Leucocyte Antigen

## Abstract

**Background:**

Relapsing polychondritis (RP) is a rare inflammatory disease characterized by recurrent inflammation and destruction of cartilaginous tissues. RP has characteristics of autoimmune disease and some reports have noted co-occurrence with autoimmune thyroid disease (AITD), consisting of Graves’ disease (GD) and Hashimoto thyroiditis (HT). However, there have been no detailed studies on the co-occurrence of RP and AITD. In this study, we aimed to determine whether patients with RP tend to be complicated with AITD. We also analyzed the clinical and genetic profiles of patients in whom these diseases co-occur.

**Methods:**

We recruited 117 patients with RP and reviewed their medical records. Furthermore, we genotyped Human Leucocyte Antigen (HLA)-A, B Cw, DRB1, DQB1, and DPB1 alleles for 93 of the 117 patients. The prevalence of AITD among the patients with RP was compared with that among the general Japanese population. We also analyzed the clinical and genetic features of the patients with both RP and AITD.

**Results:**

The prevalence of GD among the patients with RP was 4.3% (5 among 117 patients), significantly higher than that among Japanese (0.11%) (p = 2.44 × 10^–7^, binomial test). RP patients with GD tended to have nasal involvement (p = 0.023) (odds ratio (OR) 2.58) and HLA-DPB1*02:02 (p = 0.035, OR 10.41). We did not find significant enrichment of HT in patients with RP.

**Conclusions:**

Patients with RP appear to be at elevated risk of GD. Nasal involvement and HLA-DPB1*02:02 characterize the subset of RP patients with GD, which may guide attempts to characterize a distinct subtype of RP for precision medicine.

**Supplementary Information:**

The online version contains supplementary material available at 10.1186/s13023-022-02261-5.

## Background

Relapsing polychondritis (RP) is a rare inflammatory disease characterized by recurrent inflammation and destruction of cartilaginous tissues, such as external ears, nose, and airways. RP also affects joints, inner ears, eyes, and large vessels. Patients with RP sometimes produce autoantibodies against collagen type II [1]. Thus, RP has been regarded as an autoimmune disease.

While the causative factors associated with the development of RP are not well known, genetic studies have provided supportive evidence of the nature of RP as an autoimmune disease. Firestein GS et al. reported the association of Human Leucocyte Antigen (HLA)- DR4 with Caucasian patients with RP [2]. Our group previously reported that HLA class II loci are important for RP susceptibility in Japanese subjects as well [3]. These results indicate that RP susceptibility is associated with HLA class II genes more than one ethnicity.

RP is reported to co-occur with other autoimmune diseases, including Behçet’s disease (BD) [4], rheumatoid arthritis, systemic vasculitis, and autoimmune thyroid disease (AITD) [5]. However, none of the associations with RP were supported by epidemiological or genetic studies using (relatively) large sample sizes, likely due to the low prevalence of RP. For example, although BD in the setting of RP is famous as “MAGIC syndrome” [4], our previous study revealed that BD and RP did not have HLA susceptibility alleles in common with enough statistical power [3]. Thus, whether the autoimmune diseases reported to co-occur with RP are actually more frequently observed in patients with RP due to an RP-related pathogenic mechanism, or whether these diseases co-occur by coincidence, is unknown.

Identification of a disease with molecular mechanisms in common with RP would give us clues to understand the basics of RP. Through daily clinical practice with patients with RP, we noticed that patients with RP seemingly had high co-occurrence of AITD, consisting of Graves’ disease (GD) and Hashimoto thyroiditis (HT). In addition, because the symptoms of AITD such as low-grade fever, fatigue, and weight loss are also shown in the patients with RP, we wondered if the complication of AITD had been overlooked in the patients with RP. Moreover, the association of AITD with HLA has also been reported [6] and this suggests that RP and AITD may have common genetic factors. In this study, we aimed to address whether AITD is significantly more prevalent among patients with RP. Furthermore, we tried to find the clinical and genetic features which characterize the co-occurrence of RP and AITD.

## Results

### The prevalence of AITD in patients with RP

We analyzed a total of 117 patients with RP whose clinical data were available. Their detailed characteristics are indicated in Table 1.

**Table 1:** Characteristics of the 117 patients with relapsing polychondritis

Sex (male/female)	35/82
Age (years old)	54.3 ± 15.4*
Age of onset (years old)	43.2 ± 16.6*
Complication of Graves’ disease	5/117 (4.3%)
Complication of Hashimoto’s thyroiditis	6/117 (5.1%)
Organ involvement	
Auricular	92/117 (78.6%)
Nasal	56/117 (47.9%)
Tracheobronchial	66/116 (56.9%)
Audiovestibular	50/117 (42.7%)
Ophthalmic	55/117 (47.0%)
Articular	57/115 (49.6%)

*Average ± standard deviation

**Fig. 1:**
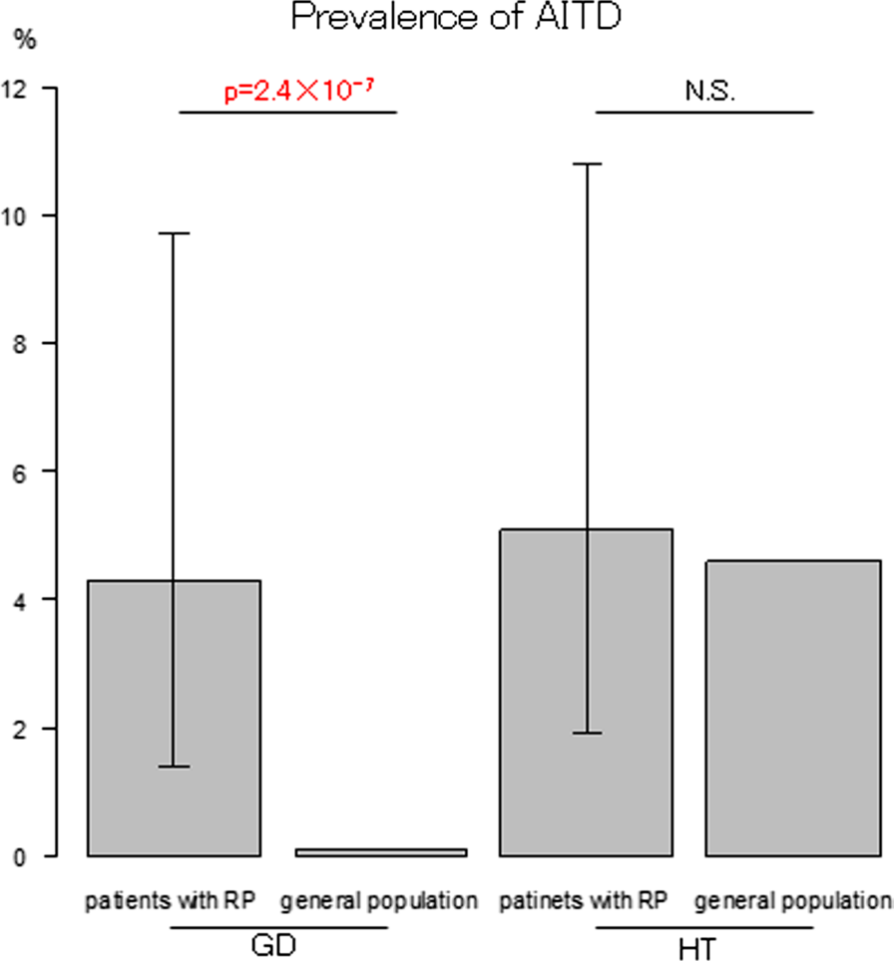
High co-occurrence rate of Graves’ disease in patients with RP. The prevalence of AITD in the patients with RP is compared with that in the general population. Bars indicate 95% confidence intervals. AITD: autoimmune thyroid disease, RP: relapsing polychondritis, GD: Graves’ disease, HT: Hashimoto thyroiditis, NS: not significant

Among the 117 patients with RP, 5 (4.3%) also carried a diagnosis of GD. The prevalence of GD among the patients with RP was significantly higher than that among the general population which was reported to be at most 0.11% [7] (p = 2.44 × 10^–7^, Fig. 1). Two of the 5 patients with RP and GD were diagnosed as RP first and the other three were diagnosed as GD first. Six (5.1%) were also diagnosed with HT. The prevalence of HT among the patients with RP was similar to that among the general population (4.6%) [8] (p = 0.66, Fig. 1).

### Clinical features of RP patients with AITD

Next, we analyzed the clinical features of the co-occurrence of RP and GD. All patients were female, which was not a significant difference from the male/female ratio of all RP patients (p = 0.32). The average age at RP onset was 49.2 ± 10.9 and 42.9 ± 16.9 in the RP patients with and without GD, respectively (p = 0.28).

Concerning the organs involved by RP, all the RP patients with GD had auricular and nasal involvements and only one of the five RP patients with GD had audiovestibular and ophthalmic involvements. RP patients with GD tended to have nasal involvement at a higher rate than RP patients without GD (100% vs 45.5%, p = 0.023, OR 2.58, [95% CI 1.09–∞], Table 2). We observed a non-significant trend of more organ involvement in the co-occurrence (3.41 ± 1.14 vs 3.18 ± 1.46), suggesting that the RP patients with GD may have the more severe and widespread disease.

**Table 2:** Differences of organ involvement between patients with and without Graves’ disease

	Patients with GD (N = 5)	Patients without GD (N = 112)	P-value	Odds ratio [95% confidence interval]
Auricular	5/5 (100%)	87/112 (77.7%)	0.58	Inf [0.26–Inf]
Nasal	5/5 (100%)	51/112 (45.5%)	0.023	Inf [1.09–Inf]
Tracheobronchial	3/5 (60%)	63/111 (56.8%)	1.00	1.14 [0.17–9.52]
Audio vestibular	1/5 (20%)	49/112 (43.8%)	0.39	0.32 [0.013–2.58]
Ophthalmic	1/5 (20%)	54/112 (48.2%)	0.37	0.27 [0.011–2.16]
Articular	2/5 (40%)	55/110 (50.0%)	1.00	0.67 [0.080–4.46]

Patients with GD tended to have nasal involvement compared with the patients without GD

P-value was obtained by Fisher’s exact test

GD: Graves’ disease

There were no clinical differences between the RP patients with HT and without HT (Additional file 1: Table S1).

### Associations between HLA alleles and co-occurrence of RP and AITD

Because classical HLA alleles are reported to be associated with the development of RP [2, 3] and AITD [6], associations between classical HLA alleles and the co-occurrence of these diseases were addressed. As a result, the frequency of HLA-DPB1*02:02 tended to be higher in patients with co-occurrence of GD and RP (20% vs 2.3%, p = 0.035, OR 10.41 [95% CI 1.23–65.38]) and none of the other HLA alleles (A, B, Cw, DRB1, and DQB1) showed even suggestive associations with AITD co-occurrence (Table 3). No HLA alleles were associated with the co-occurrence of HT (Additional file 1: Table S2). There were no distinct clinical features of the RP patients with both GD and HLA-DPB1*02:02. The average age at onset of RP was 51.0 ± 5.66 among the RP patients with both GD and HLA-DPB1*02:02 and this was slightly higher than that of the RP patients without GD (42.4 ± 16.8, p = 0.24).

**Table 3:** Human Leucocyte Antigen (HLA) alleles in patients with RP with and without GD

	Patients with GD (N = 5)	Patients without GD (N = 88)	P-value^*^	Odds ratio [95% confidence interval]
*HLA-A*				
02:01	2 (20%)	19 (10.8%)	0.31	2.01 [0.29–9.78]
02:06	1 (10%)	19 (10.8%)	1.00	0.92 [0.040–6.46]
02:07**	1 (10%)	4 (2.3%)	0.24	4.70 [0.18–40.87]
11:01	1 (10%)	27 (15.3%)	1.00	0.61 [0.027–4.12]
24:02	3 (30%)	55 (31.3%)	1.00	0.94 [0.20–3.67]
26:01	0 (0%)	12 (6.8%)	1.00	0 [0.00–6.01]
26:03	1 (10%)	4 (2.3%)	0.24	4.70 [0.18–40.87]
31:01	2 (20%)	19 (10.8%)	0.31	2.06 [0.29–9.78]
33:03	0 (0%)	14 (8.0%)	1.00	0 [0.00–5.97]
*HLA-B*				
07:02	0 (0%)	10 (5.7%)	1.00	0 [0.00–7.14]
15:01	2 (20%)	18 (10.2%)	0.30	2.18 [0.31–10.48]
35:01*	1 (10%)	18 (10.2%)	1.00	0.98 [0.043–6.93]
39:01	0 (0%)	7 (4.0%)	1.00	0 [0.00–11.88]
40:01	0 (0%)	11 (6.3%)	1.00	0 [0.00–6.51]
40:02	1 (10%)	12 (6.8%)	0.52	1.51 [0.065–11.84]
40:06	0 (0%)	4 (2.3%)	1.00	0 [0.00–21.06]
44:03	0 (0%)	10 (5.7%)	1.00	0 [0.00–7.14]
46:01*	1 (10%)	4 (2.3%)	0.24	4.70 [0.18–40.87]
48:01	0 (0%)	4 (2.3%)	1.00	0 [0.00–21.06]
51:01^†^	2 (20%)	14 (8.0%)	0.21	2.87 [0.40–14.55]
52:01	1 (10%)	16 (9.1%)	1.00	1.11 [0.049–8.07]
54:01	1 (10%)	18 (10.2%)	1.00	1.00 [0.043–6.93]
55:02	0 (0%)	6 (3.4%)	1.00	0.98 [0.00–15.36]
59:01	0/10 (0%)	5/176 (2.8%)	1.00	0 [0.00–17.83]
67:01***	1/10 (10%)	5/176 (2.8%)	0.29	3.75 [0.15–36.33]
*HLA-Cw*				
01:02	2 (20%)	33 (18.8%)	1.00	1.08 [0.16–5.03]
03:03	3 (30%)	26 (14.8%)	0.19	2.46 [0.52–9.91]
03:04	1 (10%)	22 (12.5%)	1.00	0.78 [0.035–5.36]
04:01	0 (10%)	9 (5.1%)	1.00	0 [0.00–8.21]
07:02	1 (10%)	28 (15.9%)	1.00	0.59 [0.026–3.94]
08:01	0 (0%)	10 (5.7%)	1.00	0 [0.00–7.14]
12:02	1 (10%)	17 (9.7%)	1.00	1.04 [0.046–7.46]
14:02	2 (20%)	11 (6.3%)	0.15	3.71 [0.51–20.24]
14:03	0 (0%)	10 (5.7%)	1.00	0 [0.00–7.14]
15:02	0 (0%)	4 (2.3%)	1.00	0 [0.00–21.06]
*HLA-DRB1*				
01:01	0 (0%)	9 (5.1%)	1.00	0 [0.00–8.21]
04:03	0 (0%)	6 (3.4%)	1.00	0 [0.00–15.36]
04:05	0 (0%)	31 (17.6%)	0.22	0 [0.0–2.14]
04:06	0 (0%)	9 (5.1%)	1.00	0 [0.00–8.21]
04:07	1 (10%)	2 (1.1%)	0.15	9.38 [0.30–129.97]
04:10	0 (0%)	3 (1.7%)	1.00	0 [0.00–32.00]
08:02	1 (10%)	7 (4.0%)	0.36	2.66 [0.11–20.53]
08:03	0 (0%)	11 (6.3%)	1.00	0 [0.00–6.51]
09:01	2 (20%)	22 (12.5%)	0.62	1.74 [0.25–8.14]
11:01	0 (0%)	5 (2.8%)	1.00	0 [0.00–17.83]
12:01	2 (20%)	6 (3.4%)	0.061	6.92 [0.89–38.52]
13:02	0 (0%)	9 (5.1%)	1.00	0 [0.00–8.21]
14:03*	0 (0%)	2 (1.1%)	1.00	0 [0.00–63.42]
14:05	1 (10%)	0 (0%)	0.054	Inf [0.93–Inf]
14:54	1 (10%)	8 (4.5%)	0.40	2.32 [0.096–18.37]
15:01	1 (10%)	15 (8.5%)	0.60	1.19 [0.052–8.78]
15:02	0 (0%)	16 (9.1%)	1.00	0 [0.00–5.00]
16:02***	1 (10%)	5 (2.8%)	0.29	3.75 [0.15–36.33]
*HLA-DQB1*				
03:01	2 (20%)	16 (9.1%)	0.25	2.48 [0.35–12.21]
03:02	1 (10%)	23 (13.1%)	1.00	0.74 [0.033–5.06]
03:03	3 (30%)	26 (14.8%)	0.19	2.46 [0.52–9.91]
04:01	0 (0%)	31 (17.6%)	0.22	0 [0.00–2.14]
04:02	1 (10%)	5 (2.8%)	0.29	3.75 [0.15–36.33]
05:01	0 (0%)	9 (5.1%)	1.00	0 [0.00–8.21]
05:02***	1 (10%)	12 (6.8%)	0.52	1.51 [0.065–11.85]
05:03	1 (10%)	1 (0.057%)	0.10	18.49 [0.46–751.60]
06:01	0 (0%)	27 (15.3%)	0.36	0 [0.00–2.56]
06:02	1 (10%)	15 (8.5%)	0.60	1.19 [0.052–8.78]
06:04	0 (0%)	8 (4.5%)	1.00	0 [0.00–9.71]
*HLA-DPB1*				
02:01	1 (10%)	46 (26.1%)	0.46	0.32 [0.014–2.07]
02:02	2 (20%)	4 (2.3%)	0.035	10.41 [1.23–65.38]
03:01	0 (0%)	5 (2.8%)	1.00	0 [0.00–17.83]
04:01	0 (0%)	9 (5.1%)	1.00	0 [0.00–8.21]
04:02	0 (0%)	9 (5.1%)	1.00	0 [0.00–8.21]
05:01*	5 (50%)	83 (47.2%)	1.00	1.12 [0.31–4.05]
09:01	1 (10%)	11 (6.3%)	0.50	1.66 [0.071–13.38]
13:01	1 (10%)	3 (1.7%)	0.20	6.27 [0.22–63.23]

The frequency of HLA-DPB1*02:02 was higher in the RP patients with GD than in the patients without GD

Alleles of which frequencies in general Japanese were over 2%, alleles found among the RP patients with Graves’ disease and alleles which have been reported to be associated with autoimmune thyroiditis, relapsing polychondritis, and Bechet disease were listed

P-value was obtained by Fisher’s exact test

GD: Graves’ disease, Inf: infinity

*Risk allele for Graves’ disease, **risk allele for Hashimoto thyroiditis, ***risk allele for relapsing polychondritis, ^†^risk allele for Bechet disease

We analyzed for associations between RP or GD susceptibility alleles and their co-occurrence. Lang et al. reported that HLA-DR4 is associated with RP development [2]. Our previous report showed that HLA-B*67:01, HLA-DRB1*16:02, and HLA-DQB1*05:02 are associated with the development of RP [3]. However, these alleles did not show associations with co-occurrence of GD in the patients with RP in this study (Additional file 1: Table S2). While HLA-B*35:01, B*46:01, DRB1*14:03, and DPB1*05:01 are risk alleles for GD [6], we found no associations between these alleles and GD co-occurrence in RP (HLA-B*46:01 showed a trend, Table 3).

Because our previous report showed serine at amino acid (AA) position 57 and histidine at AA position126 in HLA-DQB1 are associated with RP [3], finally, we examined whether these AAs were associated with GD development in the patients with RP. One of RP patients with GD and 12 of RP patients without GD had this combination of AA (p = 0.54) (OR 1.57 [95% CI 0.60–13.37]).

## Discussion

This study showed for the first time that patients with RP are more likely to be diagnosed with GD than the general population in Japan. Although AITD has been reported to co-occur with RP in the previous literature using a limited sample size, this co-occurrence has not been statistically shown. The data of the 117 subjects with RP, comprising more than 20% of all subjects with this diagnosis alive in Japan, enabled us to analyze the co-occurrence in a statistically robust manner. While, beyond nasal involvement by RP, we did not show common clinical features in patients with GD and RP, increasing samples size may reveal additional commonalities in auto-immune phenotype.

Although there was no national surveillance of AITD in Japanese, Akamizu et al. estimated the prevalence of GD among Japanese to be 0.073–0.11% based on the data of Japanese outpatient clinics [7]. We took the conservative way and adopted the maximum number of that report, 0.11%, as the prevalence of GD among the general population in order to avoid false positives. On the contrary, there was no estimated prevalence of HT among Japanese, we selected the regional survey carried out in Hisayama [8], which was famous for epidemiological surveys of cardiovascular disease et al. [9].

We also showed that HLA-DPB1*02:02 was suggestively associated with the co-occurrence of RP and GD. Interestingly, HLA-DPB1*02:02 had been reported to be associated with the mild phenotype of GD [10]. The symptom of GD among the patients with RP may be mild and this may lead to overlooking of GD among the patients with RP. In the present study, detailed treatment information was obtained for two patients with both RP and GD. One was treated with thiamazole and the other had the right lobe of the thyroid removed. Clinicians are encouraged to be vigilant for GD co-occurring among their patients with RP.

Detailed treatment information was obtained for 27 RP patients, 11 of whom had nasal involvement. RP patients with nasal involvement tended to receive more aggressive treatment (63.6% and 18.2% received treatment with immunosuppressive agents and biologics, respectively, compared to 31.3% and 12.5% of patients without nasal involvement). While the differences were not statistically significant, these may suggest that the RP patients with nasal involvement should be treated more aggressively. In addition, the RP patients with GD tended to have more organs affected by RP than those without GD, suggesting that the disease activity of patients with co-occurrence was more severe.

Recently, we have reported the frequent co-occurrence of Takayasu arteritis and ulcerative colitis, one of the inflammatory bowel diseases (IBD), driven by HLA-B*52:01 [11]. We also reported that treatment for IBD, ustekinumab, could be applied to the treatment for Takayasu arteritis [12]. This may indicate that if one disease commonly co-occurs with another disease and these two diseases have a common genetic background, a treatment for one disease could potentially apply to the other. This hypothesis could be applied to RP and AITD. In fact, glucocorticoids are the mainstay of the treatment for RP [13] and Graves' orbitopathy [14], which is an eye involvement of GD. Further study is necessary to analyze common basic mechanisms underlying these two diseases, which may lead to novel treatment options potentially useful for both.

There are some limitations to our study. First, this study is a retrospective study and thyroid function tests are available in a limited number of patients with RP. This prevents us from analyzing in detail the relationship between GD and RP susceptibility or its genetic components (such as whether RP patients had higher/lower thyroid function in general). Second, the number of RP patients with AITD was so small that we may fail to find important clinical or genetic features of the RP patients with AITD. Indeed, the statistical power of HLA-DPB1*02:02 to obtain a *p*-value less than 0.05 in this study was only 0.01. More patients with RP should be prospectively recruited for further studies on the association of RP and AITD to identify important clinical and genetic features characterizing the co-occurrence. Third, because we could not compare the prevalence of GD between RP and the general population using the same methods [7, 8], there might exist various potential biases such as different observational periods and evaluation of thyroid function. These biases could lead to potential overestimation or underestimation of the results.

## Conclusions

This study is the first report that patients with RP have a significantly higher prevalence of GD than general Japanese. The RP patients with GD may be characterized by the nasal involvement and inheritance of HLA-DPB1*02:02.

## Methods

### Patients

We recruited 124 patients with RP from Kyoto University Hospital, St Marianna University Hospital, and the Japanese Committee of patients with RP, the name of which is HORP (Helpful/Harmonious/Hopeful Organization for Relapsing Polychondritis). All the patients with RP fulfilled the diagnostic criteria of McAdams [15] or Damiani [16]. Seven of 124 patients were excluded from this study because of a lack of clinical information. Patients with RP complicated with AITD were fulfilled the guidelines of the Japan Thyroid Association for the diagnosis of GD and HT (http://www.japanthyroid.jp/en/guidelines.html) or diagnosed as AITD by the specialists of thyroid diseases based on clinical information. Written informed consent was obtained from each participant. This study was approved by the Kyoto University Graduate School and Faculty of Medicine, Ethics Committee, and the ethics committee of St Marianna University School of Medicine and carried out in accordance with the Declaration of Helsinki.

### Data collection of patients

We performed a retrospective chart review of 117 patients with RP in order to determine their clinical features including sex, age at this study, age at RP onset, organ involvements, and co-occurrence of AITD. Among the many potential organ involvements of RP, we scored presence or absence of involvement of six sites, namely auricular, nasal, tracheobronchial, audio-vestibular, ophthalmic, and articular involvements, which are listed in the diagnostic criteria of McAdams [15].

### HLA genotyping and analysis

We obtained blood cells or cells of buccal mucosa from 93 out of the 117 patients with RP for the genotyping of HLA. The WAKFlow system (Wakunaga) was used for genotyping of the classical HLA alleles in the six loci, namely, HLA-A, B, Cw, DRB1, DQB1, and DPB1, in the Department of Transfusion Medicine and Cell Therapy, Kyoto University Hospital. In order to obtain the data of AA position of HLA, we referred to the data of IPD-IMGT/HLA (https://www.ebi.ac.uk/ipd/imgt/hla/).

### The prevalence rate of AITD of general Japanese

Since there has been no national surveillance of the prevalence of AITD in Japan, we used the estimated Japanese prevalence of GD by Akamizu et al. [7] and the regional survey of the prevalence of HT in Hisayama [8] as the prevalence of AITD of the general population.

### Statistical analysis

Prevalence of diseases was compared using the binomial test, categorical variables were compared using Fisher’s exact test and continuous variables were compared using Welch’s t-test. P-values less than 0.05 after Bonferroni’s correction were defined as statistically significant and uncorrected p-values are indicated in the main text, figure, and tables. In the analysis of HLA, we selected alleles with frequencies of more than 2% in Japanese subjects according to the data of HLA Laboratory (http://hla.or.jp/), alleles which were found in the RP patients with AITD or alleles which are the risk alleles for RP, AITD, and BD. R 3.62 with packages “exact2 × 2” and “epi. R” was used for data analysis.

## Supplementary Information

**Additional file 1.** Differences of organ involvement between patients with and without Hashimoto thyroiditis.

## Data Availability

The datasets used and/or analyzed during the current study are available from the corresponding author on reasonable request.

## Abbreviations

RP: Relapsing polychondritis
HLA: Human Leucocyte Antigen
BD: Behçet’s disease
AITD: Autoimmune thyroid disease
GD: Graves’ disease
HT: Hashimoto thyroiditis
HORP: Helpful/Harmonious/Hopeful Organization for Relapsing Polychondritis
AA: Amino acid
OR: Odds ratio
CI: Confidence interval
