# Supplementary material for: Co-occurrence of relapsing polychondritis and autoimmune thyroid diseases
Source: Orphanet J Rare Dis. 2022 May 10;17:101. doi: 10.1186/s13023-022-02261-5 (PMC9087919; doi:10.1186/s13023-022-02261-5)
Supplement: Supplementary file 1 — Additional file 1. Differences of organ involvement between patients with and without Hashimoto thyroiditis. [file 13023_2022_2261_MOESM1_ESM.docx]

Supplementary Table S1. Differences of organ involvement between patients with and without Hashimoto thyroiditis

|  | Patients with HT  (N= 6) | Patients without HT  (N= 111) | P-value | Odds ratio  [95% confidence interval] |
| --- | --- | --- | --- | --- |
| Auricular | 4/6 (66.7%) | 88/111 (79.3%) | 0.61 | 0.53 [0.092~4.14] |
| Nasal | 3/6 (50%) | 53/111 (47.7%) | 1.00 | 1.09 [0.19~6.30] |
| Tracheobronchial | 5/6 (83.3%) | 61/110 (56.8%) | 0.23 | 3.98 [0.51~95.40] |
| Audio vestibular | 1/6 (16.7%) | 49/111 (44.1%) | 0.24 | 0.26 [0.011~1.98] |
| Ophthalmic | 1/6 (16.7%) | 54/111 (48.6%) | 0.21 | 0.21 [0.0089~1.65] |
| Arthralgia | 3/6 (50%) | 54/109 (49.5%) | 1.00 | 1.02 [0.18~5.86] |

There were no differences in the organ involvements between the RP patients with HT and without HT.

P-value was obtained by Fisher’s exact test

HT: Hashimoto thyroiditis,

Supplementary Table S2. Differences of Human Leucocyte Antigen (HLA) between patients with and without Hashimoto thyroiditis

|  | Patients with  HT (N= 5) | Patients without  HT (N= 88) | P-value | Odds ratio  [95% confidence interval] |
| --- | --- | --- | --- | --- |
| HLA-A | | | | |
| 02:01 | 1 (10%) | 20 (11.4%) | 1.00 | 0.87 [0.038~6.05] |
| 02:06 | 2 (20%) | 18 (10.2%) | 0.29 | 2.18 [0.31~10.48] |
| 02:07** | 1 (10%) | 4 (2.3%) | 0.24 | 4.70 [0.18~40.87] |
| 11:01 | 2 (20%) | 26 (14.8%) | 0.65 | 1.49 [0.21~6.70] |
| 24:02 | 3 (30%) | 55 (31.3%) | 1.00 | 0.94 [0.20~3.67] |
| 26:01 | 0 (0%) | 12 (6.8%) | 1.00 | 0 [0.00~6.01] |
| 26:03 | 1 (10%) | 3 (1.7%) | 0.20 | 6.27 [0.22~63.22] |
| 31:01 | 0 (0%) | 21 (11.9%) | 0.61 | 0 [0.00~3.53] |
| 33:03 | 0 (0%) | 14 (8.0%) | 1.00 | 0 [0.00~5.97] |
| HLA-B | | | | |
| 07:02 | 0 (0%) | 10 (5.7%) | 1.00 | 0 [0.00~7.14] |
| 15:01 | 2 (20%) | 18 (10.2%) | 0.29 | 2.18 [0.31~10.48] |
| 35:01* | 2 (20%) | 17 (9.7%) | 0.27 | 2.32 [0.33~11.29] |
| 39:01 | 0 (0%) | 8 (4.5%) | 1.00 | 0 [0.00~9.71] |
| 40:01 | 0 (0%) | 12 (6.8%) | 1.00 | 0 [0.00~6.01] |
| 40:02 | 0 (0%) | 13 (7.4%) | 1.00 | 0 [0.00~5.63] |
| 40:06 | 0 (0%) | 4 (2.3%) | 1.00 | 0 [0.00~21.06] |
| 44:03 | 1 (10%) | 9 (5.1%) | 0.43 | 2.05 [0.086~17.98] |
| 46:01* | 1 (10%) | 4 (2.3%) | 0.24 | 4.70 [0.18~40.87] |
| 48:01 | 0 (0%) | 4 (2.3%) | 1.00 | 0 [0.00~21.06] |
| 51:01^†^ | 0 (0%) | 16 (9.1%) | 1.00 | 0 [0.00~5.01] |
| 52:01 | 2 (20%) | 16 (9.1%) | 0.25 | 2.48 [0.35~12.21] |
| 54:01 | 1 (10%) | 18 (10.2%) | 1.00 | 0.98 [0.043~6.93] |
| 55:02 | 1 (10%) | 5 (2.8%) | 0.29 | 3.75 [0.15~36.33] |
| 59:01 | 0 (0%) | 5 (2.8%) | 1.00 | 0 [0.00~17.83 |
| 67:01*** | 0 (0%) | 6 (3.4%) | 1.00 | 0 [0.00~15.36] |
| HLA-Cw | | | | |
| 01:02 | 3 (30%) | 32 (18.2%) | 0.40 | 1.92 [0.41~7.58] |
| 03:03 | 2 (20%) | 27 (15.3%) | 0.66 | 1.38 [0.20~6.41] |
| 03:04 | 0 (0%) | 23 (13.1%) | 0.61 | 0 [0.00~3.14] |
| 04:01 | 1 (10%) | 8 (4.5%) | 0.40 | 2.32 [0.096~18.37] |
| 07:02 | 0 (0%) | 29 (16.5%) | 0.37 | 0 [0.00~2.33] |
| 08:01 | 1 (10%) | 9 (5.1%) | 0.43 | 2.05 [0.086~17.98] |
| 12:02 | 2 (20%) | 16 (9.1%) | 0.25 | 2.48 [0.35~12.21] |
| 14:02 | 0 (0%) | 13 (7.4%) | 1.00 | 0 [0.00~5.63] |
| 14:03 | 1 (10%) | 9 (5.1%) | 0.43 | 2.05 [0.086~17.98] |
| 15:02 | 0 (0%) | 4 (2.3%) | 1.00 | 0 [0.00~21.06] |
| HLA-DRB1 | | | | |
| 01:01 | 0 (0%) | 9 (5.1%) | 1.00 | 0 [0.00~8.21] |
| 04:03 | 0 (0%) | 6 (3.4%) | 1.00 | 0 [0.00~15.36] |
| 04:05 | 1 (10%) | 30 (17.0%) | 1.00 | 0.54 [0.024~3.60] |
| 04:06 | 1 (10%) | 8 (4.5%) | 0.40 | 2.32 [0.096~18.37] |
| 04:07 | 1 (10%) | 2 (1.1%) | 0.15 | 9.37 [0.30~129.97] |
| 04:10 | 0 (0%) | 3 (1.7%) | 1.00 | 0 [0.00~32.00] |
| 08:02 | 1 (10%) | 7 (4.0%) | 0.36 | 2.66 [0.11~20.53] |
| 08:03 | 0 (0%) | 11 (6.3%) | 1.00 | 0 [0.00~6.51] |
| 09:01 | 2 (20%) | 22 (12.5%) | 0.62 | 1.74 [0.25~8.14] |
| 11:01 | 0 (0%) | 5 (2.8%) | 1.00 | 0 [0.00~17.83] |
| 12:01 | 1 (10%) | 7 (4.0%) | 0.36 | 2.66 [0.11~20.53] |
| 13:02 | 0 (0%) | 9 (5.1%) | 1.00 | 0 [0.00~8.21] |
| 14:03* | 0 (0%) | 2 (1.1%) | 1.00 | 0 [0.00~63.42] |
| 14:05 | 0 (0%) | 1 (0.57%) | 1.00 | 0 [0.0~334.4] |
| 14:06 | 1 (10%) | 2 (1.1%) | 0.15 | 9.37 [0.30~129.97] |
| 14:54 | 0 (0%) | 8 (4.5%) | 1.00 | 0 [0.00~9.71] |
| 15:01 | 1 (10%) | 15 (8.5%) | 0.60 | 1.19 [0.052~8.78] |
| 15:02 | 1 (10%) | 15 (8.5%) | 0.60 | 1.19 [0.052~8.78] |
| 16:02*** | 0 (0%) | 6 (3.4%) | 1.00 | 0 [0.00~15.36] |
| HLA-DQB1 | | | | |
| 03:01 | 2 (20%) | 16 (9.1%) | 0.25 | 2.48 [0.35~12.21] |
| 03:02 | 3 (30%) | 21 (11.9%) | 0.12 | 3.14 [0.65~13.02] |
| 03:03 | 2 (20%) | 27 (15.3%) | 0.66 | 1.38 [0.20~6.41] |
| 04:01 | 1 (10%) | 30 (17.0%) | 1.00 | 0.54 [0.024~3.60] |
| 04:02 | 0 (0%) | 6 (3.4%) | 1.00 | 0 [0.00~15.36] |
| 05:01 | 0 (0%) | 9 (5.1%) | 1.00 | 0 [0.00~8.21] |
| 05:02*** | 0 (0%) | 13 (7.4%) | 1.00 | 0 [0.00~5.63] |
| 05:03 | 0 (0%) | 2 (1.1%) | 1.00 | 0 [0.00~63.42] |
| 06:01 | 1 (10%) | 26 (14.8%) | 1.00 | 0.64 [0.029~4.33] |
| 06:02 | 1 (10%) | 15 (8.5%) | 0.60 | 1.19 [0.052~8.78] |
| 06:04 | 0 (0%) | 8 (4.5%) | 1.00 | 0 [0.00~9.71] |
| HLA-DPB1 | | | | |
| 02:01 | 2 (20%) | 45 (25.6%) | 1.00 | 0.73 [0.11~3.39] |
| 02:02 | 0 (0%) | 6 (3.4%) | 1.00 | 0 [0.00~15.36] |
| 03:01 | 1 (10%) | 4 (2.3%) | 0.24 | 4.70 [0.18~40.87] |
| 04:01 | 0 (0%) | 9 (5.1%) | 1.00 | 0 [0.00~8.21] |
| 04:02 | 0 (0%) | 9 (5.1%) | 1.00 | 0 [0.00~8.21] |
| 05:01* | 6 (60%) | 82 (46.6%) | 0.52 | 1.71 [0.44~6.69] |
| 09:01 | 1 (10%) | 11 (6.3%) | 0.50 | 1.66 [0.071~13.38] |
| 13:01 | 0 (0%) | 4 (2.3%) | 1.00 | 0 [0.00~21.06] |

There were no association between HLA and complication of HP among patients with RP.

Alleles of which frequencies in general Japanese were over 2%, alleles found among the RP patients with Graves’ disease and alleles which have been reported to be associated with autoimmune thyroiditis, relapsing polychondritis and Bechet disease were listed.

P-value was obtained by Fisher’s exact test

HT: Hashimoto thyroiditis

*: risk allele for Graves’ disease, **: risk allele for Hashimoto thyroiditis, ***: risk allele for relapsing polychondritis, †: risk allele for Bechet disease
